# Render Unto Cæsar, Etc.

**Published:** 1875-08

**Authors:** 


Render unto Cæsar, etc.—We have received a letter from Dr. Ferdinand King, in reference to our article on "Medical Indorsement, on page 14. He informs us that in the address referred to he distinctly stated that he had drawn largely from the *Pharmacal Gazette* in its preparation, but that, by some inadvertency in preparing it for publication, this part of it was omitted. We cheerfully record the doctor's acknowledgment, regretting, with him, that due credit was not given in the published report.—*Pharmacal Gazette.*

